# Clinical Cases

**Published:** 1893-03

**Authors:** B. N. Banerjee

**Affiliations:** Calcutta, India


### CLINICAL CASES.

B. N. Banerjee, M. D., Calcutta, India.

### A Case of Typhlitis.

K. C. D., a young man, aged twenty-four years, had been suffering from obstruction of bowels from January 6th, 1889, and was under the treatment of eminent allopathic physicians for eleven days. Rectal injections with long tubes, and various other measures were adopted to move the bowels to no effect. When I was called to see the patient on January 18th, I found the patient with the following symptoms: Appearance anxious and pinched, eyes sunken, extremities cold, hiccoughs in succession of three at a time, intolerant nausea and vomiting of ingesta and a swelling about the size of an orange in the ileocæcal region which was painful and tender to touch. The right leg drawn toward abdomen and supported by a pillow underneath, no stool during the last eleven days. The least motion produced agonizing pain. The whole abdomen was distended, sore, very painful, and sensitive to touch. A little food would cause colicky pain and vomiting afterward.

A dose of Sulphur$^{200}$ was given. The next morning the hiccough was entirely gone and the patient felt greatly relieved, though the ileo-cæcal swelling remained as before. I was in a great fix—Sulphur had done some good, but he said that he felt better—so instead of prescribing any medicine I decided to wait. Up till eleven P. M. of the 19th, the patient did well, so much so that he slept for nearly three hours during the day, after five days of sleeplessness. After eleven P. M. the symptoms came back with redoubled vigor. I saw him again at twelve, when I could not trace any new symptom, and therefore prescribed another dose of Sulphur$^{200}$ and waited the whole night to see the effect of the medicine.

January 20th.—No abatement of suffering—hiccough and all other symptoms troubled the patient very much and the pulse became quite imperceptible, and a very thin, offensive discharge came out from the rectum.

Crotalus$^{200}$, one dose, was given at seven A. M. I shall never forget the magical effect it produced. The whole train of painful symptoms subsided within an hour. In the evening the patient passed a large papescent stool preceded by three or four large balls.

The patient made a rapid recovery without any repetition or new prescription, but the swelling in the ileo-cæcal region remained for some time, and disappeared gradually within a fortnight.

### A Case of Eczema Cured by Chelidonium.

A. K. D., aged twenty-eight, a lawyer by profession, came under my treatment on March 14th, 1890, for a very bad form of eczema in both his legs, from which he has been suffering for the last three years continually. He had been suffering from the following symptoms of dyspepsia since he was admitted to the bar, ten years ago:

Feeling of anguish in the pit of the stomach when it is empty and relieved by eating, stitches in the region of liver, shooting toward the back, abdomen distended after eating with rumbling, heartburn, collection and constant spitting of bitter water from the mouth, great absence of mind, forgetting the thread of argument while arguing a case, sleep disturbed by dreams of relatives long dead.

The eczema in the legs at first consisted of red and painful pimples, with a great deal of itching and oozing of a yellowish liquid. The pimples bursting from itching produced an angry-looking oozing ulcer.

Chelidonium$^{30}$, morning and evening, for four days, relieved the itching to a considerable extent. Stopped medicine for four days, but there being no further improvement Chelidonium was again continued for a fortnight, with the result that the itching, oozing, and painfulness of the parts disappeared altogether. No more medicine was given, and the patient got rid of his eczema completely.

### Cases of Hernia Cured by Oxalic Acid.

I was called to treat a case of inguinal hernia hurriedly, as the patient was in great agony. On examining the patient I found that he was suffering from strangulated inguinal hernia of the left side. He came down from up-country as the hernia remained strangulated for six days. A surgeon was called in, but he could not reduce it and therefore proposed an operation. The patient was afraid to undergo operation, and I was called in to treat him. I do not remember where I read that Oxalic acid was useful in strangulated hernia, but I am sure I read it in one of the American journals. I prescribed it in the sixth trituration, and the hernia went up after six doses of medicine. I presented it empirically. I searched all standard works, but could not find any reference to Oxalic acid in hernia. Since then I have been able to relieve several cases of strangulated hernia. It is right to state here that I have failed in three cases, which is to be expected of a medicine used empirically. The object of my bringing this case before this meeting is to learn whether any proving has been made of Oxalic acid in which any mention is made of hernia.

